# Genetic and Epigenetic Alterations of *TERT* Are Associated with Inferior Outcome in Adolescent and Young Adult Patients with Melanoma

**DOI:** 10.1038/srep45704

**Published:** 2017-04-05

**Authors:** Brittani Seynnaeve, Seungjae Lee, Sumit Borah, Yongseok Park, Alberto Pappo, John M. Kirkwood, Armita Bahrami

**Affiliations:** 1 Department of Pediatric Hematology/Oncology, Children’s Hospital of Pittsburgh of University of Pittsburgh Medical Center, Pittsburgh, Pennsylvania 15224, USA; 2 Department of Pathology, St. Jude Children’s Research Hospital, Memphis, Tennessee 38105, USA; 3 Department of Biostatistics, University of Pittsburgh, Pittsburgh, Pennsylvania 15261, USA; 4 Department of Oncology, St. Jude Children’s Research Hospital, Memphis Tennessee 38105, USA; 5 Department of Hematology/Oncology, University of Pittsburgh Medical Center, Pittsburgh, Pennsylvania 15213, USA.

## Abstract

Progression of melanoma to distant sites in adolescents and young adults (AYAs) is not reliably predicted by clinicopathologic criteria. *TERT* promoter mutations when combined with *BRAF/NRAS* mutations correlate with adverse outcome in adult melanoma. To determine the prognostic value of *TERT* alterations in AYA melanoma, we investigated the association of *TERT* promoter mutations, as well as promoter methylation, an epigenetic alteration also linked to *TERT* upregulation, with TERT mRNA expression and outcome using a well-characterized cohort of 27 patients with melanoma (ages 8–25, mean 20). TERT mRNA expression levels were significantly higher in tumors harboring *TERT* promoter mutation and/or hypermethylation than those without either aberration (*P* = 0.046). *TERT* promoter mutations alone did not predict adverse outcomes (*P* = 0.50), but the presence of *TERT* promoter methylation, alone or concurrent with promoter mutations, correlated with reduced recurrence-free survival (*P* = 0.001). These data suggest that genetic and epigenetic alterations of *TERT* are associated with *TERT* upregulation and may predict clinical outcomes in AYA melanoma. A more exhaustive understanding of the different molecular mechanisms leading to increased TERT expression may guide development of prognostic assays to stratify AYA melanoma patients according to clinical risk.

Despite stable or declining incidence rates for most types of cancer in the US, the rate of pediatric and adolescent melanoma has increased from the 1970s to 2009^1^,^2^,^3^,^4^,^5^. Although recent reports indicate a mitigation of this trend^6^,^7^,^8^, melanoma remains one of the most commonly occurring solid tumors in adolescents and young adults (AYAs) aged 15–29 years^9^,^10^,^11^, accounting for 4% of all cancers diagnosed in this age group^12^. AYAs with cancer suffer from poorer care and a lag in outcome improvements and it is unclear whether this group should be classified and treated as similarly to older adults, younger pediatric patients, or as a unique subgroup altogether^13^,^14^,^15^.

In contrast to the static situation in AYA, advances in understanding the genomics of adult melanoma have changed the treatment paradigm for advanced staged melanoma in adults. For example, approximately 50% of adult melanomas carry an oncogenic *BRAF*^V600^ mutation and approximately 20% carry an oncogenic *NRAS* mutation^16^, prompting use of selective inhibitors which target the mitogen-activated protein kinase (MAPK) and phosphatidylinositol 3-kinase-AKT pathways^17^,^18^,^19^. Mutations of the *TERT* promoter, often in combination with *BRAF* or *NRAS* mutations, also frequently occur in melanoma^20^,^21^ and correlate with poorer prognosis, adverse prognostic indicators at the primary site, and lower overall survival^22^,^23^,^24^,^25^,^26^,^27^,^28^. Other genetic and epigenetic aberrations of *TERT* have also been documented in melanoma, such as copy number amplification and promoter hypermethylation^29^,^30^,^31^.

Unfortunately it is unclear to what extent the data derived from adult melanoma studies are relevant for AYA melanoma since biological differences between age groups may influence tumor characteristics and patient outcome. A recent genomic study of 23 pediatric melanomas revealed that adolescent and adult conventional melanomas are similar in that both (i) have a high burden of ultraviolet-induced signature mutations, (ii) commonly harbor activating mutations in *BRAF* and the *TERT* promoter, and (iii) commonly harbor inactivating alterations of the *CDKN2A* and *PTEN* tumor suppressor genes^32^. Given the association of *TERT* promoter mutations with adverse outcome in adult melanoma patients^22^, we investigated the prognostic value of these mutations, as well as promoter hypermethylation− an epigenetic alteration linked to *TERT* upregulation in a subset of melanomas^29^, using 28 tissue specimens from a well-annotated cohort of 27 AYA melanomas at the University of Pittsburgh. This cohort included cases of conventional melanoma (*n* = 20), nevoid melanoma (*n* = 2), and spitzoid melanoma (*n* = 6). We also measured TERT mRNA expression levels and screened for mutations in *BRAF, NRAS* and loss of p16 expression. A subset of these samples were additionally screened for genomic rearrangement involving *TERT*. The prognostic value of these for recurrence-free survival and overall survival was then calculated.

## Results

### *TERT* Promoter Mutations

**Figure 1:**
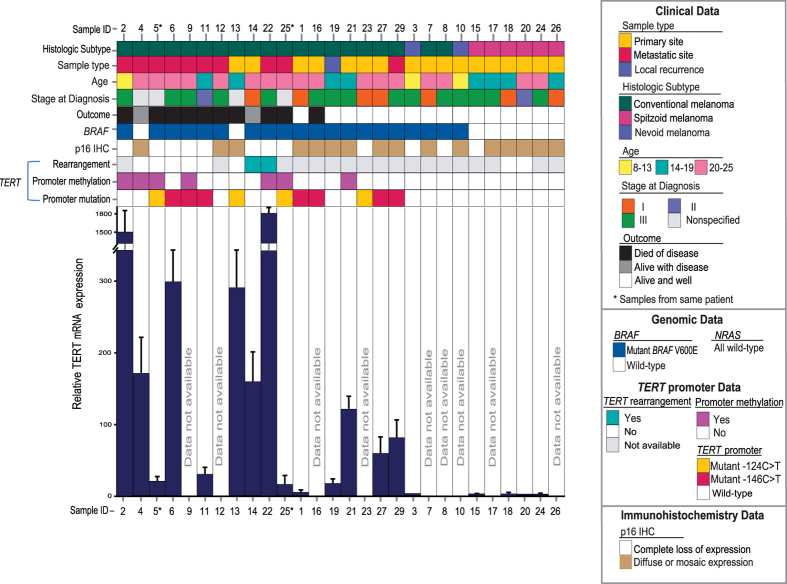
Relative TERT mRNA expression by RT-qPCR and the associated genomic, clinical, and outcome data for 28 melanoma samples from AYA patients.

Sequencing of the *TERT* promoter revealed that 10 of 19 (53%) conventional melanomas harbored promoter mutations (3 cases of −124C > T and 7 cases of −146C > T; Fig. 1). Results for the 2 samples from the same conventional melanoma patient were identical. None of the nevoid or spitzoid melanomas contained these mutations. The rs2853669 −245A > G single nucleotide polymorphism was present in 14 of 27 (52%) of all patients and in 11 of 19 (58%) cases of conventional melanoma (Supplementary Table 1).

### *TERT* Promoter Methylation Analysis

MassARRAY revealed that 8 of 19 (42%) cases of conventional melanomas and none of the nevoid or spitzoid melanomas harbored hypermethylated CpG dinucleotides in the Upstream of the Transcription Start Site (UTSS) region of the *TERT* promoter (Supplementary Table 2); hypermethylation in this region has been shown to correlate with increased TERT expression and poorer patient outcome in a number of different cancers^33^. Those samples for which the average methylation of the five UTSS CpG dinucleotides was above 15% were considered as having hypermethylated *TERT* promoter, as per Castelo-Branco *et al*.^33^. In order to additionally verify that all five of these CpG dinucleotides were methylated in cis on the same *TERT* promoter in each of these samples, the corresponding UTSS amplicon was cloned and approximately 20 clones from each sample were sequenced. For two of the samples identified as ‘hypermethylated’ by MassARRAY (ID#6 and ID#16), none of the sequenced clones harbored all five CpG dinucleotides methylated (Supplementary Figure 1). These samples were thus considered to not have hypermethylated *TERT* promoter.

### TERT mRNA Expression

RNA of a sufficiently high quality for reverse transcription and quantitative PCR (RT-qPCR) analysis was extracted from 14 of 19 conventional melanomas (8 with mutated *TERT* promoter; 6 with wild-type *TERT* promoter), 1 nevoid melanoma, and 4 spitzoid melanomas. The level of TERT mRNA expression was highly variable among the conventional melanomas and low or undetectable in the nevoid and spitzoid melanomas. TERT mRNA levels in the conventional melanomas were 4- to 300-fold (median, 69-fold) above the reference sample (spitzoid melanoma ID#20), while two of these samples (ID#2 and #22) had extremely high expression levels as compared with the other samples. Median relative TERT mRNA level was significantly higher in cases of conventional melanoma than in nevoid or spitzoid samples (*P* = 1.2 × 10^−6^) and in tumors harboring *TERT* promoter mutation and/or hypermethylation than those without either aberration (*P* = 0.046).

### *BRAF* and *NRAS* Mutations

Seventeen of the 19 (89%) conventional melanomas and both nevoid melanomas harbored the activating *BRAF*^V600E^ mutation. All 6 spitzoid melanomas had wild-type *BRAF*. No *NRAS* mutations were observed. Identical results were obtained for the 2 samples which were obtained from the same patient (Fig. 1).

### p16 Immunohistochemistry

p16 expression was assayed by immunohistochemical analysis. 10 of the 19 (53%) conventional melanomas were scored as negative for expression or as having only rarely-observed positive cells, consistent with biallelic deletions, inactivating mutations, or epigenetic alterations of *CDKN2A* in these cases. Results were similar for the 2 samples from the same patient. p16 was also undetectable in 1 of 2 (50%) nevoid melanomas and 1 of 6 (17%) spitzoid melanomas (Fig. 1).

### *TERT* Break-apart Assay

**Figure 2:**
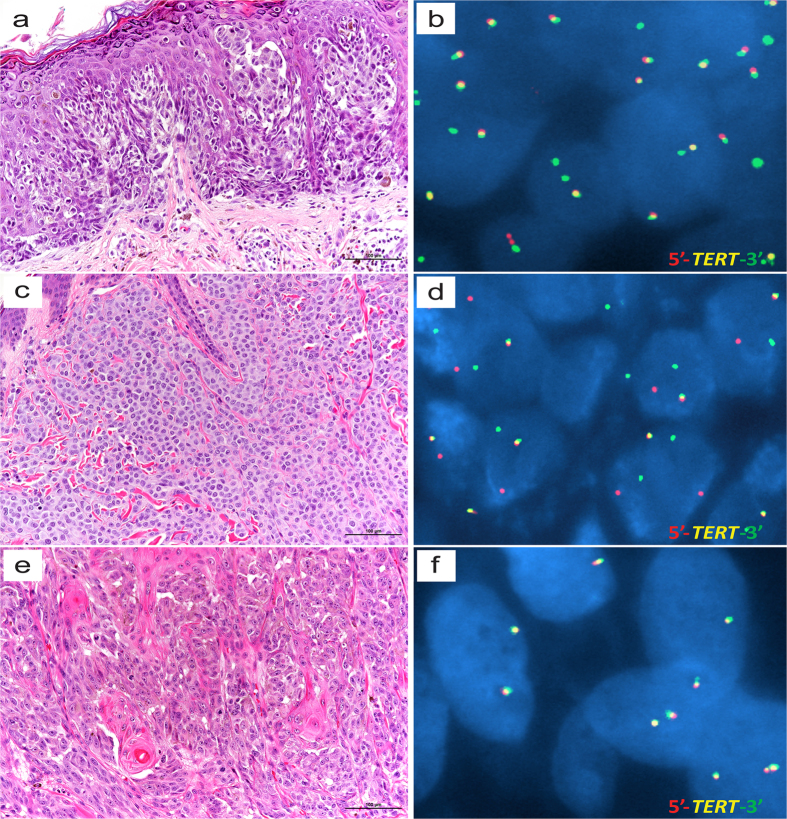
Photomicrographs of hematoxylin and eosin-stained (H&E) sections and interphase fluorescence *in situ* hybridization (FISH) with *TERT* dual color, break-apart probe from AYA patients with melanoma. (**a**,**b**) 21-year-old male (ID#14) with scalp conventional melanoma primary (H&E, 20×), alive with disease. FISH image (**b**) suggests *TERT* rearrangement with deletion of the 5′ *TERT* in most nuclei. (**c**,**d**) 20-year-old male (ID#22) with scalp conventional melanoma primary, dermal/subcutaneous metastasis (H&E, 20×), died of disease. FISH image (**d**) shows separate red and green signals in most nuclei, consistent with *TERT* rearrangement. (**e**,**f**) 23-year-old female (ID#20) with posterior trunk spitzoid melanoma (H&E, 20×), alive with no evidence of disease. FISH image (**f**) shows no break-apart (split) signals. Scale bar = 100 μm.

Fluorescence *in situ* hybridization revealed that 2 of 8 conventional melanomas tested harbored rearrangements involving the *TERT* locus (Fig. 2). Rearrangements involving *TERT* were not observed in either of the 2 spitzoid melanomas tested (Fig. 2).

### Association Analyses

*TERT* promoter mutation and/or methylation was significantly more common in conventional melanoma than in spitzoid or nevoid melanoma (*P* = 0.001). The presence of *TERT* promoter mutation and/or methylation was not associated with higher stage at presentation (*P* = 1.00), ulceration (*P* = 1.00), nodal metastasis (*P* = 1.00), or increased Breslow thickness (*P* = 0.58). TERT mRNA expression levels were significantly higher in tumors harboring *TERT* promoter mutation and/or methylation than those without either aberration (*P* = *0.046*).

Patients with melanoma harboring *TERT* promoter mutation and/or methylation had a shorter recurrence-free and overall survival than melanoma patients without these alterations, although these differences did not reach statistical significance (*P* = 0.06 and *P* = 0.08, respectively). When we analyzed the effect of *TERT* promoter mutations on survival, no correlation was found with recurrence-free or overall survival (*P* = 0.50 and *P* = 0.38, respectively). In addition, within the subgroup of melanoma patients harboring *TERT* promoter mutation, no statistically significant difference in disease-free survival was found between carriers and noncarriers of the rs2853669 −245A > G polymorphism (*P* = 0.92; Supplementary Table 1). The presence of *TERT* promoter methylation, on the other hand, alone or concurrent with promoter mutations, was significantly associated with reduced recurrence-free survival (*P* = 0.001), but not reduced overall survival (*P* = 0.06). Loss of p16 expression was not associated with either recurrence-free or overall survival (*P* = 0.26 and *P* = 0.63, respectively).

## Discussion

Our study suggests that *TERT* promoter alterations, specifically CpG dinucleotide methylation, may have prognostic value in AYA melanoma. A major challenge in treating AYA melanoma is the difficulty in recognizing histologically abnormal melanocytic lesions as unequivocally malignant, even when analyzed by the most experienced pathologists. Although overall survival of adults with melanoma correlates with the stage of disease at diagnosis, the utility of stage alone in AYAs is complicated by increasing incidence of positive lymph nodes at diagnosis in the younger population^27^,^34^,^35^. For example, Livestro *et al*. observed the 5- and 10-year survival rates of pediatric (mean age 17.2 years) and adult (mean age 53.8 years) cohorts to be similar, despite a higher prevalence of lymph node metastases in the pediatric cohort as compared with the tumor thickness-matched adult control group^36^. Likewise, an initial diagnosis of stage III disease in our cohort did not robustly predict unfavorable outcome, and in fact adverse outcomes were seen in a subset of the patients (ID#11 and ID#14) who initially presented with stage I or II disease (Fig. 1). Clinicians are thus often reluctant to treat pediatric and AYA melanoma patients in the same manner as adults with similar pathologic findings, further contributing to difficulties in managing these cases.

For these reasons, effective molecular markers could greatly aid in identifying overtly malignant AYA melanomas and improving patient care in this age group. Griewank *et al*. demonstrated that *TERT* promoter mutations independently associate with poor prognosis in primary and metastatic melanoma^22^. Populo *et al*. showed a similar association of *TERT* promoter mutations with worse outcome for primary adult melanoma^28^. Macerola *et al*. found *TERT* promoter mutations to associate with unfavorable prognostic parameters such as increasing thickness, high mitotic rate, lymph node metastasis, and presence of ulceration when *BRAF* mutations were also present, although survival analysis was not performed and no significant correlations were found between the presence of promoter mutations and regional and distant metastases^24^. Nagore *et al*. showed that coexistence of *TERT* promoter and *BRAF* or *NRAS* mutations was associated with a 2-fold reduced rate of disease-free survival and a 5-fold reduced rate of melanoma-specific survival in patients with stage I and II melanoma^26^. *TERT* promoter hypermethylation is also an effective molecular marker for prognosis in a number of cancer types, and was previously shown to correlate with increased TERT expression in a subset of melanomas^29^,^33^,^37^,^38^,^39^.

In this study we found that *TERT* promoter methylation, alone, or in combination with promoter mutations, was associated with reduced recurrence-free survival. The significance of this finding should be interpreted cautiously, however, due to the heterogeneous samples and small cohort size. In some of these cases, *TERT* promoter methylation was the sole aberration involving *TERT* observed, whereas in other cases promoter methylation occurred alongside promoter mutation or rearrangement. For samples harboring both genetic and epigenetic alterations of *TERT*, the chronological order in which these alterations occurred is not known. Matched primary, recurrent and metastatic melanoma samples would be required for this analysis.

Loss of p16 expression was also not predictive of poor prognosis in this AYA cohort, unlike in adults^40^,^41^. *TERT* promoter mutations are acquired before the loss of p16 expression in adult invasive melanoma^42^. Interestingly, however, at least two of these AYA cases had undetectable or low levels of TERT mRNA and complete loss of p16 expression in our study (ID#3 and ID#15), suggesting that the order of these events during tumor evolution may be different in a subset of AYA melanomas than in adults. Although the sample size in this study is too small to draw a meaningful conclusion, it is possible that escape from *TERT* reactivation might occur in a subset of invasive melanoma in AYA patients, and those tumors might follow a biologic course that is different from that in the adult counterpart.

An even higher incidence of *BRAF* mutation was observed in conventional AYA melanoma patients (89%) than in adults^16^, in agreement with previous studies, including our own (87%)^16^,^32^,^43^,^44^. In a population-based study of 912 cutaneous melanoma patients, higher (> = T2b) but not lower (< = T2a) stage tumors with either *NRAS* or *BRAF* mutation had a significantly poorer melanoma-specific survival rate as compared with tumors harboring wild type *NRAS* and *BRAF*, after adjusting for other prognostic factors. We found no incidence of *NRAS* mutation in this cohort and did not observe a significant correlation between *BRAF* mutation and clinical outcome, although our sample size was insufficient for subgroup analysis^44^.

TERT mRNA expression level was significantly higher in cases of conventional melanoma than in nevoid or spitzoid samples. TERT mRNA levels were also significantly higher in melanomas harboring *TERT* promoter mutation and/or methylation than in those without these alterations. However, technical difficulties in measuring TERT mRNA levels in tumor tissue limit its use as a prognostic marker. RT-qPCR assays require high-quality RNA extracted from highly pure tumor samples, which is often not possible for biopsy samples. Also, although *TERT* is silenced in the majority of somatic cells, it remains active in some non-cancerous proliferating cells such as stem cells, activated lymphocytes, and hair follicle epithelial cells. Contamination by these cells in biopsy samples can therefore prevent accurate estimation of mRNA levels in actual tumor cells.

In some of the conventional AYA melanoma cases studied here, the *TERT* promoter was neither mutated nor hypermethylated. Since the frequency of the *TERT*-independent Alternative Lengthening of Telomere mechanism in melanoma is low^45^, and since genomic rearrangement involving *TERT* is another mechanism for upregulation of this gene^46^, we tested whether a subset of the AYA cases harbored such rearrangements, including those cases which lacked mutated or hypermethylated *TERT* promoter. Indeed, two instances of *TERT* rearrangement were observed (Fig. 2); in one of these cases neither mutated nor hypermethylated *TERT* promoter was detected. Thus, understanding which of the possible genetic or epigenetic pathways for *TERT* upregulation correlates with especially high levels of TERT expression in melanoma may facilitate development of more reliable prognostic assays. Of note, a relatively high level of TERT mRNA was measured in a primary melanoma which later developed distant metastasis, despite the fact that the tumor harbored a wild-type *TERT* promoter and was initially diagnosed as stage I disease (ID #14; Fig. 1 and 2). This case highlights the danger of using only one of the multiple known DNA-level mechanisms for *TERT* dysregulation as a proxy for TERT mRNA expression level.

In summary, our study suggests that *TERT* is upregulated through various genetic and epigenetic alterations in AYA melanoma. The combination of *TERT* promoter mutation, hypermethylation and, perhaps, rearrangement, effectively serve as a diagnostic proxy for TERT mRNA levels; these DNA-level aberrations are significantly easier to detect in patient samples than mRNA levels. There are a number of limitations to our study. A relatively limited number of samples from a single institution were used in this study. In addition, the follow-up time on several patients was relatively short and the results may not reflect longer term outcome. Although a larger cohort is necessary to confirm these findings, they nevertheless highlight the need for a comprehensive understanding of the of *TERT* dysregulation in AYA melanoma to develop prognostic assays for clinical risk stratification.

## Materials and Methods

Institutional review board (IRB) approval was obtained from the University of Pittsburgh for patient enrollment in the tissue banking and analysis protocols (UPCI 96–099 and UPCI 15-202) and informed consent was obtained from all subjects. IRB approval was obtained from St. Jude Children’s Research Hospital, and studies were conducted in accordance with approved guidelines.

### Study Population

Conventional melanomas were identified in 19 patients aged 13–25 years (median, 21.0). There were 10 males and 9 females, of whom 18 were Caucasian and 1 was Indian Asian. Primary tumors arose in the skin of the trunk (*n* = 8), lower extremities (*n* = 5), scalp (*n* = 4), and unspecified site (*n* = 2). Histologic subtypes were as follows: superficial spreading (*n* = 8), nodular (*n* = 4), and not otherwise specified (*n* = 7). At last follow-up (mean, 82.9 months; range, 12–293 months), 9 patients had died of disease, 2 were alive with disease, and 8 were alive with no evidence of disease (Table 1; Fig. 1).

Nevoid melanomas occurred in 2 Caucasian males aged 8 and 13 years. The melanomas arose in the skin of the trunk (*n* = 2). Both patients were alive with no evidence of disease at last follow-up (109 months and 110 months, respectively) (Table 1; Fig. 1).

Spitzoid melanomas occurred in 6 Caucasian females aged 16–23 years (mean, 19.3; median, 18.5; standard deviation, 3.0). The melanomas arose in skin of the lower extremities (*n* = 3), upper extremities (*n* = 1), trunk (*n* = 1), and ear (*n* = 1). All patients were alive with no evidence of disease at last follow-up (mean, 49.0 months; range, 7–96 months) (Table 1; Fig. 1).

### Disease Characteristics

The stages of disease (American Joint Committee on Cancer staging system, 7^th^ edition) at diagnosis for patients with conventional melanoma were as follows: stage I (*n* = 5), stage II (*n* = 1), stage III (*n* = 10), and unspecified (*n* = 3). Of the patients with conventional melanoma who underwent sentinel lymph node evaluation at diagnosis (*n* = 16), 10 had at least 1 positive node and 6 had no nodal involvement. Of the 19 patients with conventional melanoma, 12 (63%) had an unfavorable clinical course: distant metastasis (*n* = 10), local recurrence (*n* = 4), and dead of disease in (*n* = 9) (Table 1 and Fig. 1).

The 2 patients with nevoid melanoma were initially diagnosed with the stage III disease, and neither experienced local recurrence or distant metastasis. The stages of disease at diagnosis for the 6 patients with spitzoid melanoma were as follows: stage I (*n* = 2), stage II (*n* = 1), and stage III (*n* = 3). No patients with spitzoid melanoma developed distant metastasis or local recurrence (Fig. 1). Table 1 summarizes the clinical and histopathologic features of patients.

### Tissue Specimens

The cohort comprised 27 AYA patients (mean age 20 years) with melanoma who were diagnosed between 1988 and 2014. Tissue specimens were obtained from the pathology archives of the University of Pittsburgh. Inclusion criteria were as follows: (1) lesions showing histologic features of invasive melanoma; (2) patients aged ≤25 years at the time of diagnosis; (3) availability of sufficient tissue for genomic assays; and (4) availability of patient demographic and follow-up information. The study material included 28 formalin-fixed paraffin-embedded (FFPE) tissue specimens: primary tumors (*n* = 17), lymph node metastatic tumors (*n* = 2), and distant metastatic tissues (*n* = 9, including 1 brain, 1 lung, and 7 skin/soft tissue metastatic tumors).

### Mutational Analysis of *BRAF, NRAS*, and *TERT* Promoter

FFPE tumor sections were manually dissected, with hematoxylin and eosin (H&E)–stained sections used to guide dissections, to obtain a minimum of 50% tumor purity in the material before DNA and RNA extraction, as previously described^32^. Mutational hotspots for *BRAF* (exon 15), *NRAS* (exons 1 and 2), and a portion of the *TERT* promoter (HG 19 coordinates, chr5: 1295151–1295347) were screened in genomic DNA of the 28 tumors, as previously described^32^. An additional primer set, using forward primer 5′-CCCACGTGCGCAGCAGGAC-3′ and reverse primer 5′-CTCCCAGTGGATTCGCGGGC-3′, was also used to detect rare mutations in the *TERT* promoter (HG 19 coordinates, chr5: 1295131–1295390)^23^. Results were screened by using CLC Main Workbench sequence analysis software version 6.0.2 (CLC bio, Cambridge, MA).

### *TERT* Methylation Analysis

500 ng of genomic DNA, isolated from FFPE tissue with the Maxwell^®^ 16 FFPE Plus LEV DNA Purification Kit (Promega), was processed with the EZ DNA Methylation-Gold™ Kit (ZYMO RESEARCH), according to the manufacturer’s protocol. Sodium bisulfite–treated DNA was used to amplify the *TERT* promoter, and the degree of methylation was measured by MassARRAY (Agena Bioscience) according to the method of Castelo-Branco *et al*. Data was analyzed with EpiTYPER. Commercially prepared High Methylated (>85% methylation) and Low Methylated (<5% methylation) Human Genomic DNA (EpigenDx, Hopkinton, MA), and reactions containing no DNA template, were used as controls.

The same PCR product obtained from sodium bisulfite-treated DNA of those samples identified as having hypermethylated UTSS by MassARRAY, as well as one sample identified as having an unmethylated UTSS, were further purified using NucleoSpin Gel and PCR Clean-up Kit (Macherey-Nagel), as per manufacturer’s instructions except that the PCR product was mixed with a 2x volume of 10% NTI buffer instead of a 2x volume of 100% NTI buffer. The product was eluted with 10 μL of buffer NE, of which 1 μL was incubated with 5 μL of 2x GoTaq Long PCR Master Mix (Promega) at 95 °C for 5 minutes and then at 72 °C for 15 minutes. Three microliters of this volume was used for cloning as per manufacturer’s instructions for the TOPO-TA 2.1 cloning system (Invitrogen). Positive clones were identified by blue/white selection and 24 clones from each sample were sequenced.

### *TERT* Break-apart Assay

Chromosomal rearrangements involving the *TERT* promoter were visualized by fluorescence *in situ* hybridization using BAC clones CH17-75N21 and CH17-410B01 (BACPAC Resources), as previously described^27^.

### TERT Expression by Real-time Quantitative Reverse-Transcription PCR

Total RNA was isolated from FFPE tissue sections by using the Maxwell^®^ 16 LEV RNA FFPE Purification Kit (Promega, Madison, WI) and converted to cDNA by using the SuperScript^®^ VILO cDNA Synthesis Kit (Invitrogen, Waltham, MA) according to the manufacturer’s protocol. To quantify TERT mRNA expression levels, real-time quantitative reverse-transcription PCR (RT-qPCR) was performed in triplicate using the TaqMan Gene Expression Assay (Life Technologies, Waltham, MA) with primers for *TERT* (Hs00972656_m1) and *GAPDH* (Hs02758991_g1) as the endogenous control, using the LightCycler^®^ 480 System (Roche, Indianapolis, IN) as previously described^47^. TERT expression was normalized by using a comparative control method of threshold cycles relative to GAPDH expression.

### Immunohistochemical Analysis of p16

Representative FFPE tumor blocks of 28 samples were cut in 4-μm sections and processed for immunohistochemical analysis, using an antibody directed against p16 (JC8; Santa Cruz Biotechnology, Dallas, TX), as previously described^32^. The immunohistochemical staining of p16 was recorded as follows: i) complete loss of p16 nuclear expression (rare or no positive tumor cells); ii) retained or partial loss of p16 nuclear expression (diffuse or heterogeneous/mosaic staining).

### Statistical Analysis

Association analyses between *TERT* promoter alterations and stage at presentation, melanoma type, ulceration, and nodal metastasis were performed using Fisher exact test. Association analysis between *TERT* promoter alterations and Breslow thickness was performed using t-test. The influence of immunohistochemical loss of p16 expression, *TERT* promoter mutations and *TERT* promoter methylation on patients’ survival from diagnosis of melanoma were investigated for the 27 patients. The patient who contributed 2 samples (ID#5 and ID#25) was included as only 1 subject for statistical analyses, given very similar genomic data results from the 2 samples. The survival endpoints were overall survival from diagnosis of primary melanoma to death and recurrence-free survival from diagnosis of primary melanoma to the first clinical recurrence of melanoma after definitive treatment. Cases in which the endpoint was not reached at the time of last follow-up were censored at that point. The log-rank test was applied to calculate *p*-values. Statistical analyses were performed using the survival package within R statistical analysis software (version 3.2.2).

## Additional Information

**How to cite this article**: Seynnaeve, B. *et al*. Genetic and Epigenetic Alterations of *TERT* Are Associated with Inferior Outcome in Adolescent and Young Adult Patients with Melanoma. *Sci. Rep.* **7**, 45704; doi: 10.1038/srep45704 (2017).

**Publisher's note:** Springer Nature remains neutral with regard to jurisdictional claims in published maps and institutional affiliations.

## Supplementary Material

Supplementary Materials

## Figures and Tables

**Table 1:** Clinical and histopathologic features for 27 AYA melanoma patients.

Melanoma Subtype	Sample ID	Age*	Race	Sex	Primary site	Sample site	Breslow (mm)	Ulcer	Sentinel LN metastasis	Stage*	Local recurrence	Distant metastasis	Follow-up*** (months)
Conventional Melanoma	1	21	C	F	Left shoulder	Primary	0.57	no	no	I	no	no	56
	2	13	C	M	Scalp	Scalp recurrence	1.7	no	yes	III	Yes	yes	75
	4	25	IA	M	Right leg	Soft tissue	NS	NS	NS	NS	Yes	no	65
	5/25	25	C	F	Left knee	Brain/soft tissue arm	NS	NS	NS	NS	yes	yes	205
	6	22	C	M	Scalp	Neck LN	12	yes	yes	III	no	yes	12
	7	21	C	F	Abdomen	Primary	1.2	yes	no	I	no	no	17
	8	24	C	F	Posterior trunk	Primary	1.1	no	yes	III	no	no	50
	9	25	C	F	NS	Soft tissue	NS	NS	yes	III	NS	yes	52
	11	16	C	F	Right scapula	Soft tissue	1.9	yes	no	II	no	yes	293
	12	24	C	F	NS	Lung	9	NS	yes	III	NS	yes	199
	13	18	C	M	Right leg	Primary	5.35	yes	NS	NS	no	yes	24
	14	21	C	M	Scalp	Primary	1.2	yes	no	I	no	yes	67
	16	22	C	M	Anterior trunk	Primary	3	yes	yes	III	no	yes	50
	19	19	C	F	Left thigh	Soft tissue left thigh	0.78	no	yes	III	yes	no	135
	21	16	C	M	Posterior trunk	Primary	1.82	no	yes	III	no	no	49
	22	20	C	F	Scalp	Soft tissue	NS	NS	yes	III	no	yes	44
	23	25	C	M	Posterior trunk	Primary	1	no	no	I	no	no	33
	27	25	C	M	Left calf	Primary	1.65	no	no	I	no	no	96
	29	19	C	M	Anterior trunk	Sentinel LN	0.9	no	yes	III	no	no	54
Nevoid Melanoma	3	8	C	M	Posterior trunk	Primary	3	yes	yes	III	no	no	109
	10	13	C	M	Posterior trunk	Primary	2.4	no	yes	III	no	no	110
Spitzoid Melanoma	15	16	C	F	Right foot	Primary	5.2	no	yes	III	no	no	87
	17	19	C	F	Right ear	Primary	2.4	no	yes	III	no	no	86
	18	17	C	F	Left leg	Primary	1.35	no	no	I	no	no	8
	20	23	C	F	Posterior trunk	Primary	2.9	yes	no	II	no	no	7
	24	23	C	F	Left hip	Primary	0.59	no	yes	III	no	no	96
	26	18	C	F	Right arm	Primary	1.32	no	yes	I	no	no	10

Abbreviations: C, Caucasian; IA, Indian Asian; NS, not specified; LN, lymph node; *at initial diagnosis; *** follow-up time from date of diagnosis to death or last contact.
