# Supplementary material for: Genetic and Epigenetic Alterations of TERT Are Associated with Inferior Outcome in Adolescent and Young Adult Patients with Melanoma
Source: Sci Rep. 2017 Apr 5;7:45704. doi: 10.1038/srep45704 (PMC5381111; doi:10.1038/srep45704)
Supplement: Supplementary Materials [file srep45704-s1.pdf]

Genetic and Epigenetic Alterations of *TERT* Are Associated with Inferior Outcome in Adolescent and Young Adult Patients with Melanoma

Brittani Seynnaeve, MD[1]*; Seungjae Lee, PhD[2]*; Sumit Borah, PhD[2]; Yongseok Park, PhD[3]; Alberto Pappo, MD[4]; John M. Kirkwood, MD[5]; and Armita Bahrami, MD[2]**

Supplementary Table 1. The rs2853669 single nucleotide polymorphism in 27 AYA patients with melanoma

Melanoma Subtype	*TERT* promoter rs2853669 –245A>G
**Conventional Melanoma**	
1	pos
2	neg
4	pos
5***	pos
6	pos
7	pos
8	pos
9	neg
11	neg
12	pos
13	neg
14	neg
16	neg
19	neg
21	neg
22	pos
23	pos
25****	pos
27	pos
29	pos
**Nevoid Melanoma**	
3	neg
10	neg
**Nevoid Melanoma**	
15	pos
17	neg
18	pos
20	neg
24	pos
26	neg

Supplementary Table 2. *TERT* promoter methylation analysis by MassARRAY in 28 AYA melanoma samples

Sample ID	TERT_CpG_1	TERT_CpG_2_3_4	TERT_CpG_5_6_7	TERT_CpG_8_9	TERT_CpG_10_11_12	TERT_CpG_13_14	TERT_CpG_15_16	TERT_CpG_17	TERT_CpG_18_19_20_21	TERT_CpG_22	TERT_CpG_23	TERT_CpG_24_25	TERT_CpG_26
13	13.0%	17.0%	9.0%	8.0%	NA	2.0%	16.0%	5.0%	7.0%	6.0%	2.0%	6.0%	NA
1	15.0%	16.0%	10.0%	9.0%	NA	8.0%	17.0%	4.0%	11.0%	8.0%	0.0%	8.0%	NA
8	35.0%	34.0%	24.0%	19.0%	NA	14.0%	22.0%	5.0%	16.0%	12.0%	5.0%	5.0%	NA
19	13.0%	15.0%	15.0%	9.0%	NA	5.0%	19.0%	8.0%	13.0%	9.0%	4.0%	11.0%	NA
23	32.0%	23.0%	25.0%	12.0%	NA	17.0%	29.0%	11.0%	13.0%	14.0%	4.0%	8.0%	NA
29	33.0%	25.0%	18.0%	16.0%	NA	16.0%	24.0%	7.0%	14.0%	12.0%	5.0%	13.0%	NA
11	36.0%	29.0%	22.0%	27.0%	NA	11.0%	26.0%	14.0%	16.0%	12.0%	13.0%	9.0%	NA
27	37.0%	28.0%	21.0%	22.0%	NA	8.0%	21.0%	8.0%	16.0%	12.0%	13.0%	10.0%	NA
12	33.0%	33.0%	28.0%	29.0%	NA	7.0%	20.0%	16.0%	17.0%	8.0%	13.0%	14.0%	NA
7	28.0%	24.0%	23.0%	13.0%	NA	12.0%	18.0%	12.0%	15.0%	14.0%	7.0%	14.0%	NA
14	19.0%	26.0%	26.0%	14.0%	NA	15.0%	24.0%	5.0%	17.0%	17.0%	7.0%	12.0%	NA
6	64.0%	52.0%	49.0%	34.0%	NA	32.0%	46.0%	31.0%	36.0%	15.0%	29.0%	16.0%	NA
25	20.0%	35.0%	40.0%	51.0%	NA	10.0%	30.0%	6.0%	11.0%	24.0%	19.0%	28.0%	NA
5	35.0%	41.0%	39.0%	35.0%	NA	18.0%	32.0%	12.0%	22.0%	25.0%	25.0%	26.0%	NA
2	34.0%	31.0%	24.0%	31.5%	NA	19.0%	43.5%	16.5%	26.0%	24.0%	27.5%	31.0%	NA
4	59.0%	44.0%	41.0%	45.0%	NA	37.0%	50.0%	34.0%	33.0%	26.0%	30.0%	29.0%	NA
21	72.0%	63.0%	43.0%	60.0%	NA	34.0%	46.0%	20.0%	37.0%	31.0%	30.0%	57.0%	NA
16	40.0%	50.0%	55.0%	61.0%	NA	12.0%	48.0%	8.0%	38.0%	51.0%	56.0%	19.0%	NA
22	66.0%	54.0%	38.0%	34.0%	NA	29.0%	44.0%	20.0%	28.0%	42.0%	35.0%	56.0%	NA
9	61.0%	59.0%	40.0%	74.0%	NA	47.0%	74.0%	51.0%	57.0%	73.0%	70.0%	56.0%	NA
3	2.0%	8.0%	6.0%	0.0%	NA	1.0%	15.0%	4.0%	7.0%	4.0%	0.0%	2.0%	NA
10	8.0%	8.0%	18.0%	19.0%	NA	5.0%	18.0%	3.0%	5.0%	3.0%	34.0%	7.0%	NA
17	27.0%	18.0%	8.0%	17.0%	NA	2.0%	13.0%	6.0%	9.0%	2.0%	3.0%	5.0%	NA
26	30.0%	29.0%	28.0%	21.0%	NA	15.0%	19.0%	6.0%	12.0%	10.0%	1.0%	13.0%	NA
15	19.0%	18.0%	15.0%	11.0%	NA	8.0%	26.0%	7.0%	10.0%	12.0%	5.0%	7.0%	NA
24	36.0%	21.0%	22.0%	25.0%	NA	8.0%	16.0%	7.0%	14.0%	10.0%	6.0%	9.0%	NA
18	28.0%	24.0%	15.0%	16.0%	NA	12.0%	17.0%	7.0%	14.0%	9.0%	6.0%	11.0%	NA
20	38.0%	33.0%	21.0%	22.0%	NA	26.0%	33.0%	21.0%	16.0%	10.0%	17.0%	16.0%	NA

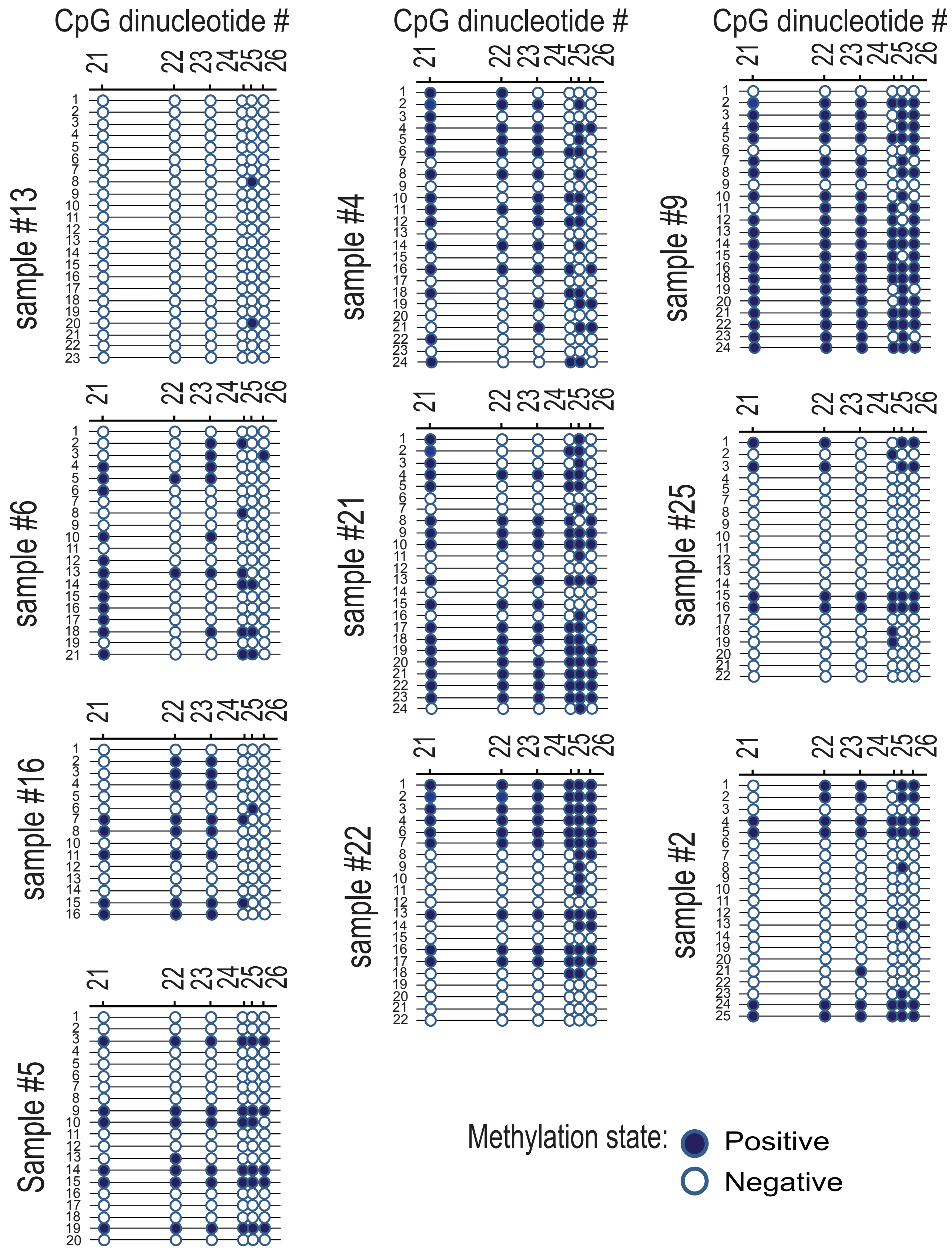

Supplementary Figure 1

Cloning and sequencing results for the Upstream of the Transcription Start Site (UTSS) region of the TERT promoter in 10 AYA melanocytic neoplasms
